# Burnout Syndrome during Residency Training in Jordan: Prevalence, Risk Factors, and Implications

**DOI:** 10.3390/ijerph18041557

**Published:** 2021-02-06

**Authors:** Abdullah Nimer, Suzan Naser, Nesrin Sultan, Rawand Said Alasad, Alexander Rabadi, Mohammed Abu-Jubba, Mohammed Q. Al-Sabbagh, Khaldoon M. Jaradat, Zaid AlKayed, Emad Aborajooh, Salam Daradkeh, Mohammad Abufaraj

**Affiliations:** 1 School of Medicine, The University of Jordan, Amman 11942, Jordan; abdulluhnimer@gmail.com (A.N.); s.naser@live.com (S.N.); Nesrin.sultan97@gmail.com (N.S.); Rawbayer@yahoo.com (R.S.A.); alexrabadi@gmail.com (A.R.); Mohamedaj203@gmail.com (M.A.-J.); 2 Medical internship, Jordan University Hospital, The University of Jordan, Amman 11942, Jordan; mqs.sabbagh@gmail.com; 3 Division of Urology, Department of Special Surgery, Jordan University Hospital, The University of Jordan, Amman 11942, Jordan; K_jaradat_80@hotmail.com; 4 Department of Psychiatry, Jordan University Hospital, The University of Jordan, Amman 11942, Jordan; zaid.alkayed@outlook.com; 5 Department of General Surgery and Anesthesia, Faculty of Medicine, Mutah University, Kerak 61710, Jordan; emad_aborajooh@yahoo.com; 6 Department of General Surgery, Jordan University Hospital, The University of Jordan, Amman 11942, Jordan; daradkeh@ju.edu.jo; 7 Department of Urology, Medical University of Vienna, 1090 Vienna, Austria

**Keywords:** burnout, Copenhagen Burnout Inventory, residency training, Jordan

## Abstract

Burnout syndrome is common among healthcare professions, including resident physicians. We aimed to assess the prevalence of burnout among resident physicians in Jordan, and a secondary aim was to evaluate the risk factors associated with the development of burnout syndrome in those residents, including gender, working hours, psychological distress, training sector, and specialty. In this cross-sectional study, 481 residents were recruited utilizing multistage stratified sampling to represent the four major health sectors in Jordan. Data were collected using an online questionnaire, where the Copenhagen Burnout Inventory (CBI) was used to assess the prevalence of burnout. The prevalence, group differences, and predictors of burnout were statistically analyzed using STATA 15. Overall, 373 (77.5%) residents were found to have burnout. Factors associated with higher levels of burnout were psychological stress (β = 2.34, CI = [1.88–2.81]), longer working hours (β = 4.07, CI = [0.52–7.62], for 51–75 h a week, β = 7.27, CI = [2.86–11.69], for 76–100 h a week and β = 7.27, CI = [0.06–14.49], for >100 h a week), and obstetrics/gynecology residents (β = 9.66, CI = [3.59–15.73]). Conversely, medical sub-specialty residents, as well as private and university hospital residents, had lower burnout levels. We concluded that decreasing the workload on residents, offering psychological counseling, and promoting a safety culture for residents might help in mitigating burnout consequences.

## 1. Introduction

The concept of burnout was introduced independently by Maslach and Freudenberger in the mid-1970s [1] and defined as a syndrome of physical, mental, and emotional exhaustion that arises in response to continuous stress [2]. The Copenhagen Burnout Inventory (CBI) uses three domains to evaluate burnout, which are personal burnout, work-related burnout, and client-related burnout [1].

Burnout frequently occurs in individuals who are working in professions involving human services, including healthcare providers [3,4]. A recent study in a teaching hospital in Jordan showed that around half of the residents suffered from a high degree of emotional exhaustion, with 8 out of 10 residents exceeding a 24-h shift length [5]. Among physicians in the United States, the prevalence of burnout is estimated to be around 50% [6], with other studies suggesting higher numbers [7]. Multiple factors were found to have a possible association with burnout in the literature, including long working hours, younger age, and higher levels of occupational stress [8,9]. Various psychiatric disorders may contribute to burnout, such as depression, anxiety, and alcohol or drug abuse [3,10,11]. Work-related stress and its consequences on mental and physical health have devastating effects, not only on physicians themselves, but also on patients and the institutions [11]. In fact, patients receiving care by physicians suffering from burnout are at higher risks of suboptimal care and medical errors, such as prescription errors [7,12].

Resident doctors are especially vulnerable to burnout due to the nature of their work [11]. They are exposed to sleep deprivation, high workloads and stress levels, unsatisfactory salaries, and a stringent work hierarchy [7,11]. Moreover, younger residents experience more risk of burnout due to the potential pressure of senior colleagues. Younger residents who are exposed to new work environments and responsibilities are especially vulnerable, taking into account the potential pressure from senior colleagues [13].

In Jordan, doctors have to undergo six years of medical school and a one-year internship before becoming eligible for residency training. During the residency program, working hours can reach around 100 hours per week [14]. In addition to spending four to six years of medical residency, residents have to sit for the Jordanian Board examinations to be eligible to practice as specialists. The Jordanian Board examinations usually consist of two parts, one testing the doctor’s general knowledge of medical and clinical sciences in a multiple-choice question (MCQ) fashion and the second assessing knowledge specific to the specialty via MCQ, oral, and clinical assessments [15]. After passing these final exams, the residents are qualified to practice as specialists throughout the country. Specialists trained in other countries have to pass these exams as well in order to be eligible to practice in Jordan.

In recent years, burnout has been exacerbated due to increasing peer competition, greater workloads, and the high expectations placed on resident doctors [12]. Failure to anticipate and prevent burnout among residents might lead to drastic consequences on healthcare. There is a scarcity of high-quality, nationwide studies assessing burnout in residents in Jordanian and Middle Eastern hospitals. In addition, the literature fails to provide a comprehensive understanding of burnout in all of healthcare as it only investigates the phenomenon in specific specialties [16]. Therefore, this study aims to assess the prevalence of burnout among medical residents in Jordan and to evaluate different factors associated with it.

## 2. Materials and Methods

### 2.1. Setting

Jordan is an upper-middle-income developing country where healthcare is mainly provided by four major sectors: the Ministry of Health (30 hospitals), Royal Medical Services (12 hospitals), the private sector (68 hospitals), and university hospitals (2 hospitals: Jordan University Hospital and King Abdullah University Hospital) [17]. Only hospitals that provide a residency training program were included in this study. The residency program in Jordan lasts 4–6 years depending on specialty [15]. Residents are either paid (“those receiving a salary”) or unpaid (“those uncompensated for their work”) [14]. The unpaid program is as an option for candidates who wish to pursue their training in a particular specialty, but their credentials did not meet the minimum requirements for that program. The timeframe for data collection was from September 2020 to October 2020.

### 2.2. Sampling and Recruitment

This was a cross-sectional study that utilized multistage stratified sampling to recruit participants from the four major health sectors in Jordan. Our population was doctors enrolled in residency training programs in Jordan, which were estimated to be 3395 residents according to the Jordan Medical Council. Each sector had a sample approximately proportional to its size (probability proportional to size—PPS): 1211 (36%) residents in the Ministry of Health, 1069 (31%) residents in the Royal Medical Services, 682 (20%) residents in university hospitals, and 433 (13%) residents in the private sector. To reach a statistical power of 0.8 and a margin of error of 5% with a confidence interval of 95%, the sample size should be at least 346. We collected 481 total responses: 174 residents from the Ministry of Health, 129 from the Royal Medical Services, 111 from university hospitals, and 67 from the private sector. Chief residents or contacts in these selected sectors were contacted by emails and social media accounts and were encouraged to disseminate the survey to their colleagues in a systematic approach.

### 2.3. Instrument Development

Data were collected using a self-administered online questionnaire. The questionnaire was developed and validated by five investigators to measure burnout and its associated factors. The Copenhagen Burnout Inventory (CBI) was used to measure burnout syndrome [1]. The CBI is comprised of three sub-dimensions: personal burnout, work-related burnout, and client-related burnout. It was found to have very high reliability and validity, as well as low non-response rates [1]. The tool most often used in the literature was the Maslach Burnout Inventory (MBI), but after reviewing the psychometric properties of both tools, we decided to use the CBI for various reasons [3]. The MBI is made up of three independent measures: emotional exhaustion, depersonalization, and reduced personal accomplishment, which cannot be joined to make up an overall score, whereas such a combination is possible using the CBI. It has also been suggested that the “Personal Accomplishment” dimension develops independently from the other two dimensions proposed by the MBI and may not be part of burnout syndrome as it is a consequence of long-term stress. Furthermore, some MBI questions may be viewed as unacceptable, such as those on depersonalization, which caused overall negative reactions among respondents [1]. For these reasons, the CBI was ultimately chosen for the purposes of our study. Out of a maximum score of 100, a score of 50 or more indicated a moderate degree of burnout, while a score of 75 or more indicated a high degree of burnout [1]. To measure psychological distress, the Patient Health Questionnaire for Depression and Anxiety (PHQ-4) was used [18]. It was shown to be a validated brief screening tool for both anxiety and depression. It also showed a strong association with functional impairment [18]. Our tool also covered other factors that might be associated with burnout (gender, specialty, training sector, payment status, workload, psychological distress, and residency year). A pilot study was conducted on 35 participants who were not included in the final study. Cronbach’s alpha value showed good reliability for PHQ-4 (0.80), excellent reliability for the overall CBI (0.95), excellent reliability for personal burnout (0.91), good reliability for work-related burnout (0.87), and excellent reliability for client-related burnout (0.92).

### 2.4. Data Analysis

Data were entered in an Excel spreadsheet and all statistical analysis was conducted using STATA 15 (Stata Statistical Software: Release 15. College Station, TX: StataCorp LLC; USA). The associations between demographic variables and the PHQ-4 scale were evaluated using the chi-square test, with statistically significant results defined as *P*-value < 0.05. Linear regression analysis was used to assess the association between different factors and the degree of burnout. Variables were first evaluated using univariate linear regression analysis. Then, significant variables were fitted into the final linear regression model.

### 2.5. Ethical Consideration

This study was approved by the Institutional Review Board (IRB) at the University of Jordan. Informed consent was taken from all participants prior to filling out the questionnaire. Participants’ privacy was preserved in this study by dealing with the data anonymously.

## 3. Results

### 3.1. Characteristics of the Sample

Table 1 shows the characteristics of the study participants and the prevalence of total burnout among them. The majority of participants were 20–25 years of age, men (55.5%), single (63.2%), working in the Ministry of Health (36%), in a residency program for surgical and sub-surgical specialties (46.6%), and in a paid program (71.5%), with an average monthly income of 501–750 Jordanian Dinars (JOD). Of all respondents, 373 (77.5%) were found to have burnout (moderate (n = 295, 61.3%), severe (n = 78, 16.2 %)). A hundred and eight residents (22.5%) did not have burnout. Males were found to have significantly higher burnout levels than females (*p* = 0.02). Burnout levels were significantly different among specialties (*p* < 0.01), residency years (*p* < 0.01), working hours (*p* < 0.01), on-calls per month (*p* < 0.01), as well as responsibility for households (*p* = 0.02).

### 3.2. Burnout Levels

Table 2 shows the mean values with the standard deviation for each question in the CBI, each category, and the total. Personal-related had the highest score of 63 ± 21, followed by work-related with a score of 61 ± 18, and the lowest was patient-related, which had a score of 55 ± 23. Overall, a total score of 59 ± 18 showed moderate burnout in residents largely due to personal feelings and work environment-related factors.

### 3.3. Factors Associated with Burnout

Table 3 shows the results of the linear regression analyses. PHQ-4 was significantly associated with burnout (β = 2.34, CI = [1.88–2.81], *p* < 0.000). Burnout was significantly higher in participants who worked more than 50 h weekly (β = 4.07, CI = [0.52–7.62], *p* = 0.025 for 51–75 h a week, β = 7.27, CI = [2.86–11.69], *p* = 0.001 for 76–100 h a week, and β = 7.27, CI = [0.06 – 14.49], *p* = 0.048 for more than 100 h a week). Compared to internal medicine, residents in obstetrics and gynecology (Ob/Gyn) had significantly higher levels of burnout (β = 9.66, CI = [3.59–15.73], *p* = 0.002), whereas other specialties (forensic medicine, psychiatry, dermatology, and pathology) showed significantly lower levels of burnout (β = −11.76, CI = [−16.39–−7.13], *p* < 0.000). Compared to residents at the Ministry of Health, university hospital residents (β = −5.49, CI = [−9.03–−1.96], *p* = 0.002) and private sector residents (β= −4.89, CI = [−9.10–−0.68] *p* = 0.023) had significantly lower levels of burnout. No significant difference in the levels of burnout was found among different age groups, genders, or residency years. Sensitivity analysis using ordinal logistic regression analysis for burnout categories showed comparable regression coefficient values to the linear regression analysis for burnout scores.

## 4. Discussion

In this representative sample of residents in Jordan, about eight out of every ten participants suffered from some degree of burnout. Psychological stress and more working hours were significantly associated with higher levels of burnout. Residents in private or university hospitals and those undergoing medical sub-specialties had lower burnout levels. The highest burnout levels were observed in residents undergoing Ob/Gyn. 

### 4.1. Total Prevalence of Burnout

We observed relatively high levels of burnout among Jordanian residents, in which about eight out of ten participants reported some degree of burnout, with a total mean burnout score of 59. All three domains were found to have a mean score of more than 50 (63 for personal-related, 61 for work-related, and 55 for patient-related burnout). These numbers are noticeably higher than those reported in the previous literature, which states that burnout prevalence among healthcare providers in the Middle East ranged from 40% to 60% [19]. The majority of articles in the previous literature utilized the MBI, making it difficult to compare our results with the literature. Studies that defined overall burnout as burnout in at least one of burnouts’ categories (84% in UAE [20] and 70% in Saudi Arabia [21]) had higher values of prevalence than studies that defined overall burnout as the presence of burnout in all three categories (11.7% in Yemen [22], 16% in Qatar [23], and 6.3% in Saudi Arabia [24]). The prevalence of burnout in Jordan was also higher than in Western countries (40% in America [16] and 75% in Canada [25]). These findings could be explained by the nature of the medical residents’ training system in Jordan and the fact that many residents choose to undergo unpaid residency to get into specialties that are highly competitive, and that most of them work around 100 h weekly [14]. Moreover, violence against physicians is a common phenomenon seen in the region that might contribute to burnout [26]. It is worth mentioning that such discrepancies could be simply due to using the CBI rather than the MBI as in our study. The CBI focuses on fatigue and exhaustion, which follows the definition of burnout more closely, but the MBI assesses burnout from different perspectives using a comprehensive approach and thus might have higher sensitivity in detecting burnout. These findings warrant instant actions by healthcare policies and a thorough understanding of factors linked to burnout.

### 4.2. Burnout Predictors

Psychological distress, measured by PHQ-4, was found to be significantly associated with burnout. According to Maswadi et al., the prevalence of anxiety and perceived stress in Jordanian resident doctors was higher than that of the normal population [27]. A study performed on Jordanian medical students also showed higher rates of exhaustion and disengagement [28]. Our results are similar to these studies and show the urgent need to address the issue of stress among medical residents. Previous literature suggested that self-administered psychotherapeutic techniques and a self-care workshop might be beneficial in decreasing workplace stress [16]. Burnout and psychological stress might be two faces of the same coin; they share many risk factors, manifestations, and complications. Therefore, promoting safety culture and offering counseling services to provide residents with coping skills and psychological resilience would be very helpful in reducing both, and subsequently improving the quality of healthcare [29].

Increased workload is significantly associated with burnout levels, as burnout scores were proportionally increasing with increased working hours. This matches the previous literature that listed workload as a risk factor for both burnout and perceived stress [12,25,30]. This suggests that reducing weekly working hours might be an appropriate solution to reduce the severity and prevalence of burnout [31]. However, reducing working hours might negatively affect the quality of training [32].

Residents in the Ob/Gyn program were found to have significantly higher levels of total burnout than residents in internal medicine and medical sub-specialties (radiology, emergency medicine, and family medicine). This can be explained by the excessive workload on Ob/Gyn residents and the frequent on-calls. Such findings can be seen as a call for program directors to accept more residents in Ob/Gyn in order to decrease their workload. On the other hand, specialties like forensic medicine, dermatology, psychiatry, and pathology were found to have significantly less total burnout than internal medicine and medical sub-specialties. In fact, the lower number of on-calls and the decreased workload compared to other specialties provide a plausible explanation for these observations. Rodrigues et al. reported similar findings in a systematic review and meta-analysis comparing burnout between medical specialties, stating that the prevalence of burnout was higher in Ob/Gyn and surgical specialties than in internal medicine and pediatrics [11]. However, a meta-analysis by Low et al. in 2019 showed no statistically significant difference in the prevalence of burnout among specialties [12].

Participants affiliated with the Ministry of Health were found to have significantly higher levels of burnout than residents in university hospitals or residents in the private sector. This can be explained by the fact that most of the Jordanian population are insured in the Ministry of Health’s institutions, putting more pressure on the residents working there, which is reflected as increased workloads and subsequently more burnout. A study conducted in Malaysia found no differences in the level of stress among doctors working in the different sectors. Moreover, doctors in the private sector were found to have more stress regarding patient care and professional uncertainty [33]. However, the authors evaluated practicing physicians and did not analyze residents specifically. Indeed, different roles and responsibilities among the two groups preclude generalization.

### 4.3. Limitations

The major limitation of our study emerges from its cross-sectional nature, and hence, a cause–effect relationship between the risk factors evaluated and burnout syndrome cannot be determined. Moreover, using an online questionnaire might result in reporting bias from social desirability or fear from discussing such sensitive topics, as direct interaction offers more trust and empathy. Nevertheless, the major strengths of this study are the stratified sampling technique and using the CBI as a data collection tool; the former was designed to represent all residents across different health sectors in Jordan, and the latter has high validity and reliability in measuring different domains of burnout.

## 5. Conclusions

The prevalence of total burnout among Jordanian residents was found to be high, reaching 77%. Psychological distress, working hours, specializing in Ob/Gyn, as well as working in the Ministry of Health sector were found to be predictors for higher levels of burnout. We recommend the following measures to address burnout among residents: covering residents with mental health insurance, holding wellness and job safety awareness days, increasing job benefits like sick leave and vacations, activating night float systems, recruiting more residents in specialties with a higher workload, and promoting a safety culture where residents are aware of their workplace rights. Future research should evaluate the complications of burnout and the economic burden and explore possible solutions that might decrease burnout.

## Figures and Tables

**Table 1:** Sociodemographic characteristics of 502 residents according to the levels of burnout as assessed by the Copenhagen Burnout Inventory.

Variable	Category	Total n (%)	No Burnout (%)	Moderate Burnout (%)	Severe Burnout (%)	*p*-Value *
Total		481 (100)	108 (22.5)	295 (61.3)	78 (16.2)	
Gender	Male	267 (55.5)	47(43.5)	176 (59.7)	44 (56.4)	0.02
	Female	214 (44.5)	61(56.5)	119(40.3)	34(43.6)	
Marital status	Unmarried	304(63.2)	73 (67.6)	182(61.7)	49(62.8)	0.55
	Married	177 (36.8)	35 (32.4)	113 (38.3)	29 (37.2)	
Training sector	Ministry of Health	174 (36)	31(28.7)	110(27.3)	33(42.3)	0.48
	University hospitals (JUH or KAUH)	111 (23)	26(24.1)	71(24.1)	14(17.9)	
	Private sector	67 (14)	18 (16.7)	40 (13.6)	9 (11.5)	
	Royal Medical Services	129 (27)	33(30.6)	74 (25.1)	22 (28.2)	
Specialty	Internal medicine and sub-medical specialties	136 (28.3)	30 (27.8)	87(28.7)	24 (28.3)	<0.01
	General surgery and sub-surgery	224 (46.6)	44 (40.7)	145 (49.2)	34 (43.6)	
	Pediatrics	39 (8.1)	4 (3.7)	25 (8.5)	10 (12.8)	
	Obstetrics and gynecology	26 (5.4)	0(0)	17 (5.8)	9 (11.5)	
	Others (forensic medicine, psychiatry, dermatology, and pathology)	56 (11.6)	30 (27.8)	23 (7.8)	3 (3.8)	
Residency year	First and second	247 (51)	48 (44.4)	146 (49.5)	53 (67.9)	<0.01
	Third and fourth	234 (49)	60 (55.6)	149(50.5)	25 (32.1)	
Paid program	Yes	344 (71.5)	75 (69.4)	213 (72.2)	56 (71.8)	0.86
	No	137 (28.5)	33(30.6)	82 (27.8)	22 (28.2)	
Salary (JOD/month)	Less than 500	22 (6.5)	5 (6.7)	14 (6.7)	3(5.5)	0.81
	501–750	237 (69.9)	50 (66.7)	151 (72.2)	36 (65.5)	
	751–1000	79 (23.3)	20 (26.7)	43 (20.6)	16 (29.1)	
	More than 1000	1(0.3)	0(0)	1(0.5)	0(0)	
Working hours per week	Less than 50	113 (23.5)	53 (49.1)	48 (16.3)	12 (15.4)	<0.01
	51–75	205 (42.6)	43 (39.8)	144 (48.8)	18 (23.1)	
	76–100	139(28.9)	11 (10.2)	88 (29.8)	40 (51.3)	
	More than 100	24(5)	1 (0.9)	15 (5.1)	8 (10.3)	
On-calls per month	5 and less	189 (39.3)	75 (69.4)	100 (33.9)	14 (17.9)	<0.01
	6–10	236 (49.1)	29 (26.9)	169 (57.3)	38 (48.7)	
	11–15	49 (10.2)	4 (3.7)	21 (7.1)	24 (30.8)	
	More than 15	7(1)	0(0)	5 (1.7)	2 (2.6)	
Responsible for households	Yes	187 (38.9)	52 (48.1)	113 (38.3)	22 (28.2)	0.02
	No	294 (61.1)	56 (51.9)	182 (61.7)	56 (71.8)	

* Bold: Significant *p*-value.

**Table 2:** The responses of 502 residents to CBI items.

Domain	Questions	Mean ± SD
Personal Burnout	Total	63 ± 21
	How often do you feel tired?	74 ± 24
	How often do you feel physically exhausted?	69 ± 26
	How often do you feel emotionally exhausted?	65 ± 26
	How often do you think “I can’t take it anymore”?	56 ± 27
	How often do you feel worn out?	60 ± 25
	How often do you feel weak and susceptible to illness?	53 ± 25
Work-Related	Total	61 ± 18
	Is your work emotionally exhausting?	65 ± 27
	Do you feel burnt out because of your work?	66 ± 26
	Does your work frustrate you?	61 ± 26
	Do you feel worn out at the end of the working day?	67 ± 25
	Are you exhausted in the morning at the thought of another day at work?	67 ± 27
	Do you feel that every working hour is tiring for you?	61 ± 26
	Do you have enough energy for family and friends during leisure time?	40 ± 26
Patient-Related	Total	55 ± 23
	Do you find it hard to work with patients?	51 ± 27
	Do you find it frustrating to work with patients?	52 ± 28
	Does it drain your energy to work with patients?	54 ± 28
	Do you feel that you give more than you get back when you work with patients?	62 ± 29
	Are you tired of working with patients?	53 ± 27
	Do you sometimes wonder how long you will be able to continue working with patients?	56 ± 29
Total Score		59 ± 18

**Table 3:** Multivariable linear regression analysis assessing factors associated with CBI burnout levels.

	Subgroups	β Coefficient	*p*-Value *	95% Confidence Interval
Age		−0.14	0.661	[−0.78–0.50]
PHQ-4		2.34	0.000	[1.88–2.81]
Gender	Male(ref)			
	Female	−2.04	0.159	[−4.89–0.80]
Specialty	Internal medicine(ref)			
	Surgical specialties	0.43	0.791	[2.75–3.61]
	Pediatrics	2.75	0.310	[−2.56–8.05]
	Ob/Gyn	9.66	0.002	[3.59–15.73]
	Other	−11.76	0.000	[−16.39–−7.13]
Year	Junior(ref): First and second years			
	Seniors: Third year and above	−0.84	0.592	[−3.93–2.24]
Working Hours per Week	50 or less(ref)			
	51–75	4.07	0.025	[0.52–7.62]
	76–100	7.27	0.001	[2.86–11.69]
	>100	7.27	0.048	[0.06–14.49]
No. of on-Calls	5 and less(ref)			
	6–10	2.08	0.228	[−1.31–5.46]
	11–15	6.33	0.020	[1.00–11.65]
	>15	6.00	0.277	[−4.83–16.83]
Sector	Ministry of Health(ref)			
	University hospitals	−5.49	0.002	[−9.03–−1.96]
	Private sector	−4.89	0.023	[−9.10–−0.68]
	Royal Medical Services	−1.96	0.254	[−5.33–1.41]

* Bold: Significant *p*-value.

## Data Availability

The data presented in this study are available on request from the corresponding author.
